# Incarcerated Spigelian Hernia: A Case Report

**DOI:** 10.7759/cureus.111549

**Published:** 2026-06-26

**Authors:** Arym P Preza Estrada

**Affiliations:** 1 General Surgery, Hospital General, Instituto de Seguridad y Servicios Sociales de los Trabajadores del Estado (ISSSTE), La Paz, MEX

**Keywords:** abdominal wall hernia, hernia incarceration, hernioplasty, mesh repair, spigelian hernia

## Abstract

Spigelian hernia (SH) is a rare abdominal wall defect that frequently presents a diagnostic challenge because of its non-specific symptoms and deep anatomical location. We report the case of a 64-year-old woman with adequately controlled hypertension who presented with localized abdominal wall pain and a long-standing lateral abdominal bulge. Physical examination revealed an incarcerated SH without signs of strangulation or bowel obstruction. Computed tomography confirmed a Spigelian fascial defect containing incarcerated ileal loops without evidence of bowel compromise, allowing appropriate preoperative planning. The patient underwent elective open hernioplasty with polypropylene mesh placement without intraoperative complications. Postoperative recovery was uneventful, with satisfactory wound healing and progressive return to normal daily activities. At six months of follow-up, the patient remained asymptomatic, with no evidence of recurrence or mesh-related complications.

This case highlights the importance of maintaining a high index of suspicion for SH in patients presenting with lateral abdominal wall pain or swelling. Computed tomography remains a valuable diagnostic tool for confirming the diagnosis and facilitating surgical planning, while timely mesh repair represents a safe and effective treatment option that may prevent progression to incarceration, strangulation, and bowel compromise.

## Introduction

Spigelian hernia (SH) is a rare abdominal wall defect resulting from a weakness or disruption of the Spigelian fascia, through which intra-abdominal contents may protrude, representing approximately 0.12%-2% of all abdominal wall hernias [1]. Despite its rarity, SH remains clinically important because of its relatively high risk of incarceration and strangulation. It develops along the semilunar line, an area of inherent weakness of the abdominal wall. The Spigelian fascia transitions to the posterior sheath of the rectus abdominis at the arcuate line, creating the so-called Spigelian belt, where increased intra-abdominal pressure further predisposes to hernia formation [1,2].

The characteristically narrow neck of SHs is associated with a risk of incarceration and strangulation of up to 20% at the time of diagnosis [3]. The clinical diagnosis of SH is often challenging; although ultrasound can aid in the evaluation of suspected SH, contrast-enhanced computed tomography is often the preferred method for confirming the diagnosis, especially in patients with equivocal clinical presentations [2].

Both open and minimally invasive approaches have been described for the treatment of SHs [2]. We present this case to emphasize the diagnostic challenges of SH, particularly in patients with longstanding abdominal wall symptoms, and to highlight the importance of computed tomography and timely surgical repair in preventing incarceration, strangulation, and bowel compromise.

## Case presentation

A 64-year-old woman with a medical history significant for well-controlled hypertension and a body mass index of 30 kg/m² presented with localized right abdominal wall pain (visual analog score: 4/10), intermittent nausea, and a long-standing bulge in the right lateral abdominal wall that had been present for approximately five years. She denied vomiting, constipation, or other symptoms suggestive of bowel obstruction. The patient had no history of smoking, substance abuse, or previous abdominal surgery.

On physical examination, a palpable right lateral abdominal wall mass measuring approximately 10 × 15 cm was identified. The lesion became more prominent with the Valsalva maneuver and was non-reducible, consistent with an incarcerated abdominal wall hernia. No fascial defect could be clearly palpated on examination. There were no overlying skin changes, signs of peritoneal irritation, or clinical evidence of bowel obstruction.

Preoperative laboratory evaluation, including complete blood count, renal function tests, and coagulation profile (prothrombin time, activated partial thromboplastin time, and international normalized ratio), revealed no significant abnormalities (Table 1).

**Table 1:** Preoperative laboratory values aPTT, activated partial thromboplastin time; BUN, blood urea nitrogen; Cr, creatinine; HCT, hematocrit; HGB, hemoglobin; INR, international normalized ratio; PT, prothrombin time.

Laboratory study	Lab value	Reference range
HGB	13.5	11.6-15 g/dL
HCT	38	36%-48%
BUN	12	6-24 mg/dL
Cr	0.8	0.6-1.2 mg/dL
PT	12	11-13.5 s
INR	1	0.8-1.2
aPTT	31	25-35 s

As part of the preoperative evaluation, a non-contrast abdominal computed tomography scan was performed, demonstrating a Spigelian fascial defect containing omentum and incarcerated ileal loops without evidence of bowel ischemia, strangulation, or obstruction. Although the original imaging study was no longer available for retrieval at the time of manuscript preparation because of institutional archiving policies, the radiological findings were documented in the medical record and subsequently confirmed during surgery.

**Figure 1:**
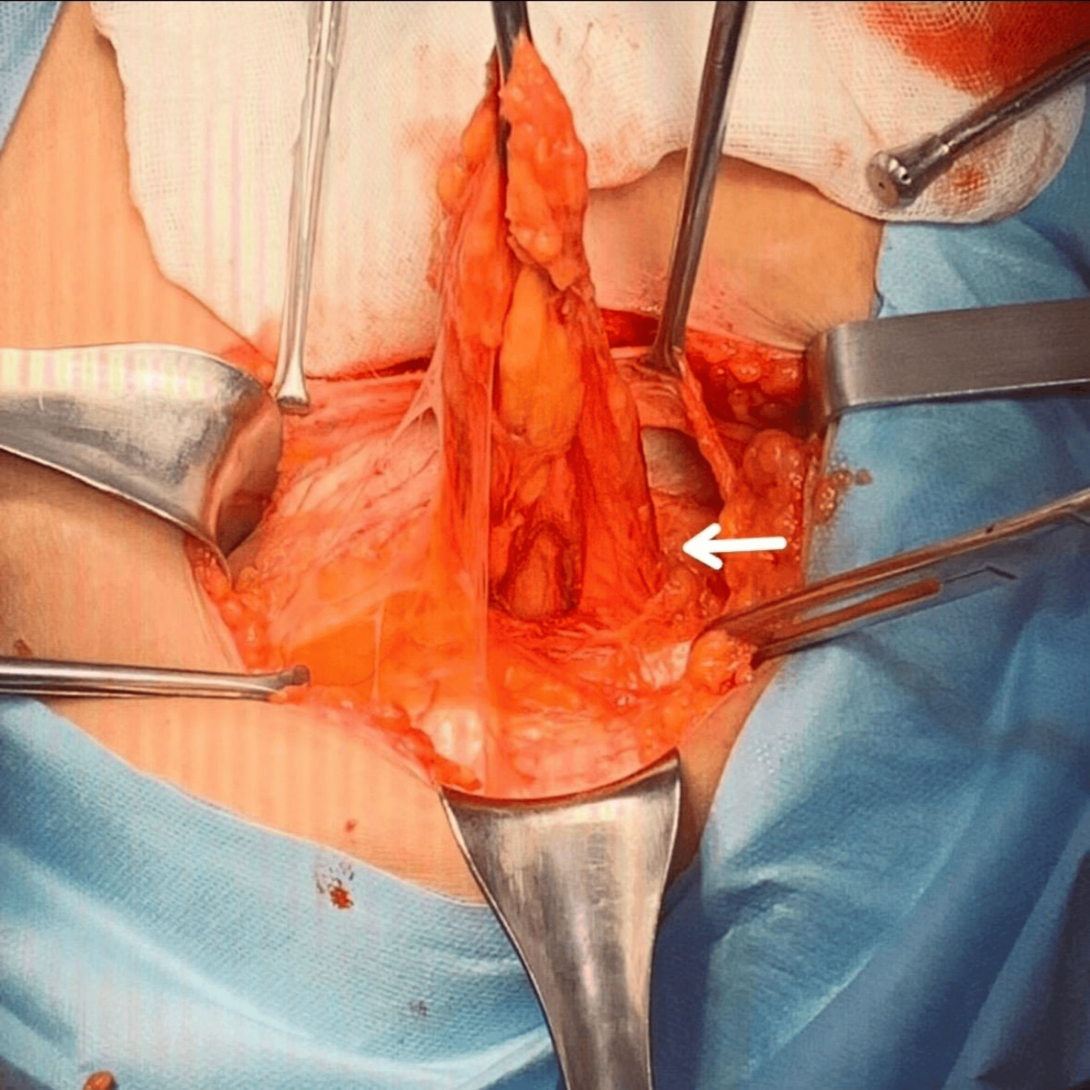
Intraoperative view of an incarcerated Spigelian hernia The white arrow indicates the hernia defect and ring, with protrusion of omentum and viable incarcerated ileal loops through the fascial defect. No signs of ischemia or strangulation were identified.

Given the persistent symptoms, chronic incarceration, and the potential risk of future strangulation, the patient was scheduled for elective surgical repair. An open hernioplasty was performed under general anesthesia. Intraoperatively, a 7-cm fascial defect was identified, associated with a hernia sac measuring approximately 10 cm in diameter and containing omentum and viable ileal loops (Figure 1).

Following reduction of the hernia contents, a 10 × 10 cm polypropylene mesh was placed in a supra-aponeurotic position and secured with interrupted 2-0 polypropylene sutures. No bowel compromise was identified, and no intraoperative complications occurred.

The postoperative course was uneventful, with adequate pain control, tolerance of oral intake, and no evidence of early postoperative complications. During outpatient follow-up, the patient demonstrated satisfactory wound healing and progressive return to her usual daily activities. At six months of follow-up, she remained asymptomatic, with no evidence of recurrence or mesh-related complications.

## Discussion

SH is a rare abdominal wall defect whose low incidence and frequently subtle clinical presentation often result in delayed diagnosis, allowing the condition to remain unnoticed for years until symptoms or complications arise. This diagnostic challenge was exemplified in the present case. 

There are two types of SH: those located above the inferior epigastric vessels, known as superior SH, and those located caudally to these vessels, referred to as inferior SH [4]. This anatomical classification is clinically relevant because the relationship of the defect to the inferior epigastric vessels and surrounding muscular layers may influence both presentation and surgical planning. Clinically, SH may present in three different forms: a small interstitial hernia measuring 1-2 cm in diameter and covered by the external oblique aponeurosis, making palpation difficult and clinical diagnosis challenging; a larger hernia measuring 3-20 cm in diameter that displaces or penetrates the superficial aponeurotic plane and becomes apparent beneath the skin; and an inflammatory mass associated with bowel obstruction or peritonitis, which occurs in cases of strangulation or phlegmon formation [5].

According to Katsaros et al., the most widely accepted theory regarding the development of SH was originally proposed by Zimmerman et al. in 1944 and attributes the condition to musculoaponeurotic defects within the transversus abdominis aponeurosis, resulting in weakness of the transversus and internal oblique muscle layers [6].

Katsaros et al. reported that the typical patient with SH is female, overweight (body mass index approximately 28 kg/m²), and in the sixth decade of life or older [6]. The characteristics of our patient are consistent with those reported in previous studies. She was a 64-year-old overweight woman with a symptomatic SH, further supporting the demographic profile commonly associated with this condition. The main symptom is pain, although nausea, vomiting, and alterations in bowel habits may also occur depending on the contents of the hernia sac. On physical examination, approximately 64% of patients present with an abdominal mass in the region of the Spigelian aponeurosis. Furthermore, up to 20% of cases present with incarceration or strangulation [7].

The relatively high rate of incarceration and strangulation reported in the literature supports early surgical intervention once the diagnosis has been established. Only 50% of cases are diagnosed preoperatively. Radiologic evaluation plays a key role in patients with suspected SH. Dynamic ultrasound may improve detection by allowing examination of the abdominal wall during changes in posture and increases in intra-abdominal pressure. Nevertheless, computed tomography remains the imaging technique most commonly used to confirm the diagnosis because of its superior ability to delineate the defect and characterize the hernia contents. In selected situations, oral contrast administration can aid in identifying bowel involvement, while magnetic resonance imaging may provide complementary information for surgical planning [8].

In the present case, computed tomography played a key role in confirming the diagnosis, defining the characteristics of the defect, and facilitating appropriate surgical planning. The variable clinical manifestations of SH may mimic other intra-abdominal and abdominal wall disorders. The differential diagnosis includes acute appendicitis, appendiceal abscess, abdominal wall neoplasms, spontaneous rectus sheath hematoma, and acute diverticulitis [8]. 

Our patient had experienced a progressively symptomatic lateral abdominal wall bulge for approximately five years before definitive diagnosis. This prolonged clinical course illustrates the diagnostic difficulties associated with SH and highlights how the condition may remain unrecognized for extended periods because of its non-specific presentation. Despite the presence of incarceration, there were no clinical or radiological signs of bowel obstruction, ischemia, or strangulation. These findings allowed elective rather than emergency surgical treatment and supported a more individualized management strategy.

Current European Hernia Society (EHS) recommendations do not establish the superiority of either open or minimally invasive repair. Consequently, the selection of the surgical approach is at the discretion of the operating surgeon [9]. Current evidence supports mesh reinforcement as the preferred repair strategy for most SHs, although primary tissue repair may be appropriate for selected small defects. Recurrence following surgical treatment is uncommon, especially after mesh-based repair. In procedures involving intraperitoneal mesh placement, the use of composite prostheses may help minimize postoperative adhesions. Adequate overlap of the prosthetic material beyond the fascial defect is generally recommended to enhance long-term repair integrity. However, the role of routine defect closure before mesh implantation remains controversial and is not specifically addressed in the current EHS guidance [10].

One advantage of laparoscopic repair is the possibility of inspecting the abdominal wall comprehensively, which may facilitate identification of additional defects that are not clinically apparent, whereas a standard open repair may require a longer incision, particularly when the hernia is not palpable [10]. Nevertheless, the choice between open and laparoscopic repair should be individualized according to patient characteristics, hernia complexity, available resources, and surgeon expertise. When performed by experienced surgeons, both approaches provide safe and effective outcomes.

Laparoscopy is considered an excellent approach for elective repair. It should generally include polypropylene mesh placement, either in an intraperitoneal or extraperitoneal position, as several studies have reported no significant differences between these techniques, with low recurrence rates of approximately 8% [11]. Although minimally invasive techniques are increasingly adopted, open mesh repair remains a safe and effective alternative. In the present case, the presence of an incarcerated hernia, the surgeon’s expertise in abdominal wall reconstruction, and the lack of laparoscopic equipment at our institution supported the decision to perform an open repair.

Hanzalova et al. reported that most patients who required emergency surgery presented with incarcerated hernia contents, and several underwent bowel resection because of the severity of their presentation. In this particular context, the decision to perform a mesh repair versus direct suture must be taken after considering the risk of wound infection [10]. Primary tissue repair should be reserved for cases with significant local infection in order to avoid the placement of prosthetic material in contaminated tissues [12]. Unlike many patients requiring emergency intervention, our patient showed no evidence of strangulation or bowel compromise, which allowed definitive mesh repair under elective conditions and may have contributed to the favorable postoperative outcome observed.

The main disadvantages of the open approach are related to wound complications, including hematoma, infection, seroma formation, and mesh infection. In addition, recurrence rates of up to 13% at six months, increased postoperative pain, and longer hospital stays have been reported [7]. Despite these potential disadvantages, open mesh hernioplasty resulted in an uneventful recovery and no evidence of recurrence during follow-up, supporting its continued use as a dependable surgical approach for SH.

## Conclusions

The present case highlights the diagnostic challenges associated with SH, a condition that may remain unrecognized for prolonged periods because of its non-specific clinical presentation. Accurate preoperative imaging facilitated diagnosis and surgical planning, and elective open mesh repair was associated with a favorable outcome in this patient. Early recognition and timely surgical management remain important to reduce the risk of incarceration, strangulation, and other potentially serious complications.
